# Supplementary material for: Sphingosine Kinases at the Intersection of Pro-Inflammatory LPS and Anti-Inflammatory Endocannabinoid Signaling in BV2 Mouse Microglia Cells
Source: Int J Mol Sci. 2023 May 9;24(10):8508. doi: 10.3390/ijms24108508 (PMC10217805; doi:10.3390/ijms24108508)
Supplement: Supplementary file 1 [file ijms-24-08508-s001.zip › ijms-2310187-supplementary.pdf]

Supplementary Materials

Sphingosine kinases at the intersection of pro-inflammatory LPS and anti-inflammatory endocannabinoid signaling in BV2 mouse microglia cells

Sara Standoli [1,†], Cinzia Rapino [2,†], Camilla Di Meo [1], Agnes Rudowski [3], Nicole Kämpfer-Kolb [3], Luisa Michelle Volk [3], Dominique Thomas [4,5], Sandra Trautmann [4,5], Yannick Schreiber [5], Dagmar Meyer zu Heringdorf [3,*] and Mauro Maccarrone [6,7,*]

1 Department of Bioscience and Technology for Food Agriculture and Environment, University of Teramo, 64100 Teramo, Italy; sstandoli@unite.it (S.S.)
2 Department of Veterinary Medicine, University of Teramo, 64100 Teramo, Italy; crapino@unite.it
3 Institute of General Pharmacology and Toxicology, University Hospital Frankfurt, Goethe University Frankfurt, 60590 Frankfurt am Main, Germany; rudowski@med.uni-frankfurt.de (A.R.); n.kolb@em.uni-frankfurt.de (N.K.-K.); volk@med.uni-frankfurt.de (L.M.V.)
4 Institute of Clinical Pharmacology, Goethe University Frankfurt, 60590 Frankfurt am Main, Germany; dominique.thomas@itmp.fraunhofer.de (D.T.); trautmann@med.uni-frankfurt.de (S.T.)
5 Fraunhofer Institute for Translational Medicine and Pharmacology (ITMP), Fraunhofer Cluster of Excellence for Immune Mediated Diseases (CIMD), 60596 Frankfurt am Main, Germany; yannick.schreiber@itmp.fraunhofer.de
6 Department of Biotechnological and Applied Clinical Sciences, University of L'Aquila, 67100 L'Aquila, Italy
7 European Center for Brain Research (CERC), Santa Lucia Foundation, Scientific Institute for Research, Hospitalization and Healthcare, 00143 Rome, Italy
* Correspondence: heringdorf@med.uni-frankfurt.de (D.M.z.H.); mauro.maccarrone@univaq.it (M.M.)
† These authors contributed equally to this work.

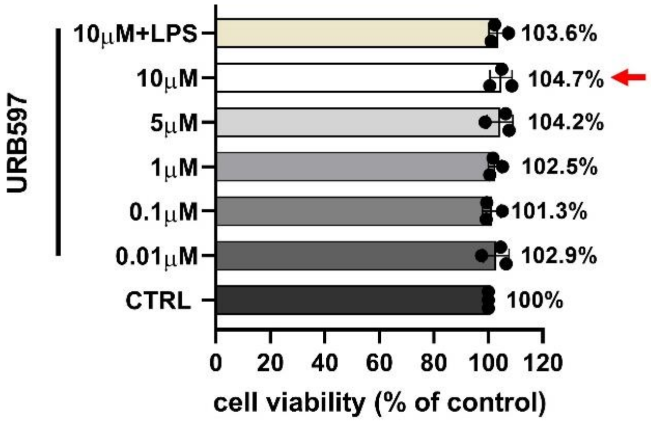

Figure S1. Assessment of cell viability by MTT assay on BV2 cells treated with URB597 at different doses (0.01, 0.1, 1.0, 5.0 and 10µM) and 10 µM URB597 together with 100ng/mL LPS, for 4h. The experimental dose used in the subsequent experiments by a red arrow. Values are expressed as % of control cells and presented as mean SEM of three independent experiment (n = 3).

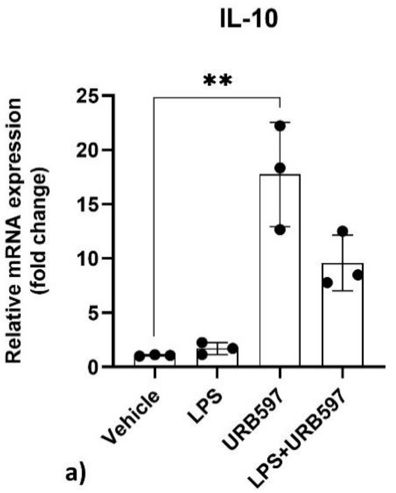

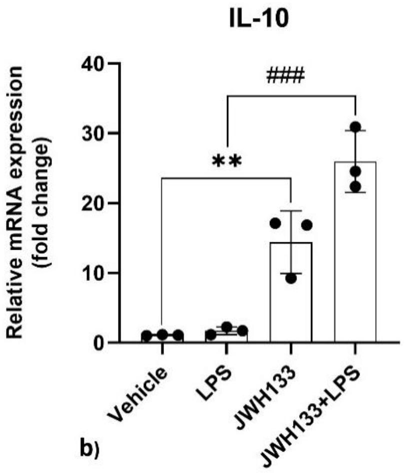

Figure S2. Effect of a) URB597 and b) JWH133, on IL-10 gene expression, in absence and in presence of LPS. BV2 cells were pre-treated with vehicle (CTRL) or 10 μM URB597/1 μM JWH133, for 30 minutes, and then further incubated with vehicle or 100 ng/mL LPS for 4 h. The mRNA level of IL-10 was determined by qRT-PCR and expressed as fold change, using the 2-ΔΔCT method. Data are means±SEM of three independent experiments each in a) and b). Statistical analysis was performed by two-way ANOVA followed by Tukey's multiple comparisons test [**p<0.01 vs. CTRL; ###p<0.001 vs. LPS].

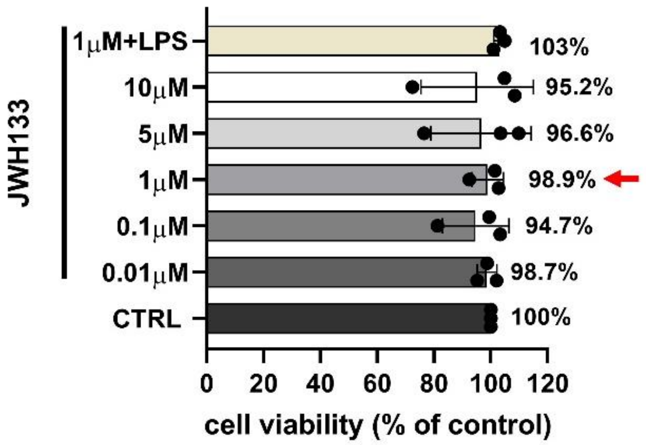

Figure S3. Assessment of cell viability by MTT assay on BV2 cells treated with JWH133 at different doses (0.01, 0.1, 1.0, 5.0 and 10μM) and 1 μM JWH133 together with 100ng/mL LPS, for 4h. The experimental dose used in the subsequent experiments by a red arrow. Values are expressed as % of control cells and presented as mean SEM of three independent experiment (n = 3).
